# Application of tight-fitting half-facepiece breath-response powered air-purifying respirator for internal body cooling in occupational environment

**DOI:** 10.1371/journal.pone.0266534

**Published:** 2022-04-06

**Authors:** Shingo Sekoguchi, Hajime Ando, Kazunori Ikegami, Hidetaka Yoshitake, Chikage Nagano, Akira Ogami

**Affiliations:** 1 Department of Work Systems and Health, Institute of Industrial Ecological Sciences, University of Occupational and Environmental Health, Kitakyushu, Fukuoka, Japan; 2 Department of Health Policy and Management, Institute of Industrial Ecological Sciences, University of Occupational and Environmental Health, Kitakyushu, Fukuoka, Japan; Universiti Teknologi Malaysia, MALAYSIA

## Abstract

In dust-generating scenarios in occupational environments, it is important to take measures to prevent not only pneumoconiosis, but also heatstroke. The aim of this study was to verify whether using a tight-fitting half-facepiece breath-response powered air-purifying respirator (PAPR) in combination with a self-produced cooling device could abate the deep body temperature while performing activities. We conducted a crossover study involving 10 subjects. The subjects were subjected to three conditions: wearing a PAPR equipped with a cooling device, PAPR, or a replaceable particulate respirator. During the experiment, the rectal temperature of the subjects was measured, along with the temperature near the PAPR inlet in container with the cooling device when the PAPR equipped with the cooling device was worn. The subjects rested in a cold chamber set at a dry-bulb temperature of 28°C and relative humidity of 45% for 20 min. Then, they moved to a hot chamber set to a dry-bulb temperature of 36°C (with the same relative humidity) in 5 min and exercised on a cycle ergometer for 30 min. After that, the subjects moved to the cold chamber for 5 min and rested for 20 min. Notably, the air inhaled by the subjects wearing PAPR equipped with the cooling device was approximately 10°C cooler than the ambient air. Furthermore, 35 min after the initiation of the experiment (after the middle of the exercise period), the rectal temperature of the participants wearing the PAPR equipped with the cooling device was lower than of those wearing PAPR or replaceable particulate respirators (*p* <0.05). Thus, we could deduce that the self-produced cooling device was useful in abating deep body temperature. PAPR is useful for its potential applications in hot occupational environments and can save lives in working environments where heat stress can result in major medical complications.

## 1. Introduction

Workers who are exposed to extreme temperatures or work in hot indoor and outdoor environments, or are engaged in high-effort physical activities, may be at risk of heat stress [1]. Those at risk of heat stress include outdoor workers and those working in high-temperature environments, such as construction and factory workers, and miners [1]. While high environmental temperatures, especially in warm regions like Japan, result in increased body heat at rest, additional heat production from physical activities further increases body heat storage in these environments. Continuing physical activity in such environments may result in severe heat-related injuries or even death [2]. In 2018, the Japan Ministry of Health, Labour and Welfare published the 13th Occupational Safety & Health Program, which aimed to promote the prevention of heatstroke in the workplace [3]. However, in the last 10 years, from 2012 to 2021, an average of 638 workers per year died or were absent from work for more than 4 days in Japan due to heatstroke at work [4], which remains relatively high.

There are four main approaches to resolving unacceptable levels of heat stress in occupational management: modifying the environment, work, or worker body condition by heat acclimatization, or providing auxiliary body cooling by altering the clothing and equipment [1]. Environmental modifications include increasing ventilation, reducing the temperature of a radiant heat source, shielding the workers, and implementing air conditioning [1, 5]. Heat stress can also be mitigated by reducing the exposure time or temperature and the metabolic heat load [1, 5]. However, the environment cannot be easily modified due to technical or economic reasons.

Some forms of cooling, before or during work, can aid in alleviating thermal stress during work under high-temperature conditions [2]. A worker may use external or internal cooling methods. External cooling involves the application of a cold medium or material to the body’s surface, and can include exposure to cold air and water, or wearing cold garments [6]. In the industrial working environment, garments equipped with cooling materials, such as ice, phase change materials, and cooled water-perfusion systems, have been developed [7–9]. Also, a long-sleeve jacket that is similar to work clothing but has small fans attached to the back of the waist has sometimes been used among workers [10–12]. Internal cooling involves the introduction of a cold medium to the body through the mouth (and/or nose, in the case of breathing), and includes the consumption of cold fluids or ice and inhalation of cold air [6]. In recent years, ingestion of ice slurry before working has attracted attention as a practical precooling method and has been reported to suppress the elevation of body temperature [2, 13–15].

Cold air inhalation may benefit individuals who experience increases in metabolic and environmental heat gain during activities at high ambient temperatures [6]. In a previous study, subjects breathing 3.6°C air during exercise in hot humid conditions (dry-bulb temperature of 38°C and relative humidity of 90–95%) could abate an increase in the deep body temperature [16]. In another study, subjects breathing 10°C air during exercise in hot conditions (dry-bulb temperature of 38°C and relative humidity of 50%) were also reported to abate an increase in the deep body temperature [17]. However, the feasibility of internal cooling by cold-air inhalation in the field has not yet been sufficiently evaluated [6].

Apart from occupational heat stress, in the field of occupational safety and health management, occupational exposures are also associated with dusty and airborne particulates that are produced excessively or unintentionally from the processing machines or devices. Respiratory protective devices (RPDs) are often used by workers in workplaces where they may be exposed to hazardous substances and dust to prevent pneumoconiosis. The use of RPDs is mandatory in Japan in dust-generating scenarios in occupational environment, as specified in the Ordinance on Prevention of Hazards Due to Dust [18] and include the replaceable particulate respirator (RPR) and disposable particulate respirator [19]. Powered air-purifying respirators (PAPRs) are also commonly used in Japan, which can be described as respirators that protect the user by filtering out contaminants in the air; they include a battery-operated blower to provide clean air to the user through a tight-fitting respirator and a loose-fitting hood or helmet [20]. In recent years, the tight-fitting half-facepiece breath-response PAPR often has been used in Japan [21, 22].

Our proposed cooling method is an extension of PAPRs, providing cold air inhalation through the PAPR for internal body cooling. Although the thermal sensations and comfort of PAPR have already been studied [23], its application to internal cooling has not been reported. We have developed a cooling device that can be attached to the PAPR to investigate its application to internal cooling. In this study, we aimed to verify whether internal cooling using a tight-fitting half-facepiece breath-response PAPR equipped with a self-produced cooling device could abate the rise in deep body temperature during work hours in demanding heated work environments.

## 2. Methods

### 2.1 Study design and settings

In this study, we conducted a crossover study comprising of 10 human subjects. A crossover study comprises controlled experiments in which participants receive different interventions sequentially. The experiment was conducted at the University of Occupational and Environmental Health, Japan, between October 1 and December 16, 2019.

### 2.2 Participants

The subjects were recruited from the University of Occupational and Environmental Health, Japan. The eligible participants were men aged ≥20 years. The examiner (one of the authors of this study), who is a doctor, interviewed the subjects before the experiment to confirm that they were healthy, with no present illness. The mean age, height, body weight, and body mass index of the subjects were 25.9±7.4 y, 170.8±4.0 cm, 70.2±11.4 kg, and 24.0±3.0, respectively.

### 2.3 Respiratory protective device

The chosen PAPR model for testing was a BL–321S (Koken Ltd., Japan). This PAPR was tight-fitted with a half-facepiece and breath-response system. The PAPR specifications are as follows: the motor blower capacity was of the large airflow volume type (≥138 L/min), leakage rate was B-class (≤5.0%), and the filtering efficiency was PL 1 (≥95.0%). PL is the PAPR, which was tested for particle collection efficiency using di (2-ethylhexyl) phthalate (DEHP) as the test particle. The breath-response system had an electronically controlled mechanism that synchronizes the airflow rate of the fan with the wearer’s breathing rate [24]. The chosen RPR model for testing was a 1180–05 (Koken Ltd., Japan), consisting of a half facepiece and a single filter with efficiency specification of RL 2 (≥95.0%). RL is the RPR, which was tested for particle collection efficiency using DEHP as the test particle. The PAPR and RPR used in this experiment were similar in shape and material, and met the standards defined by the Japanese Ministry of Health, Labour and Welfare [25, 26].

### 2.4 Cooling device

The cooling device used in this experiment was developed by the authors and has a patent-pending (Japanese Unexamined Patent Application Publication No. 2021–108963).

**Fig 1:**
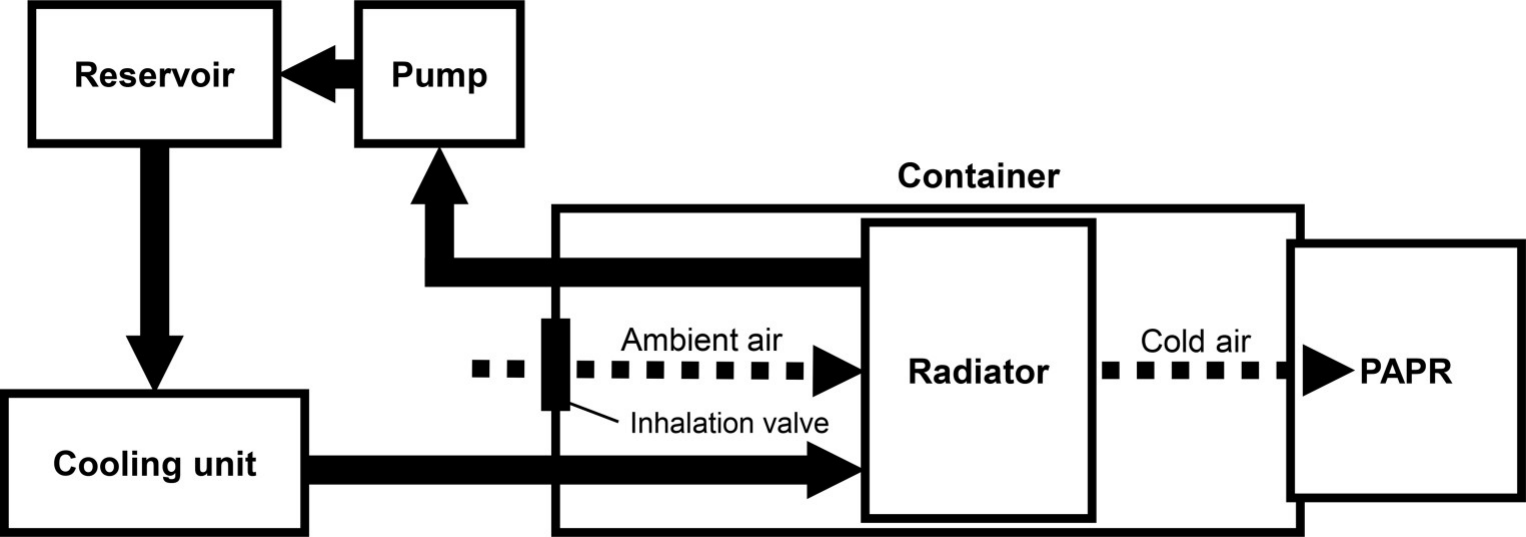
Diagram of the cooling device developed in this study. PAPR: powered air-purifying respirator.

**Fig 2:**
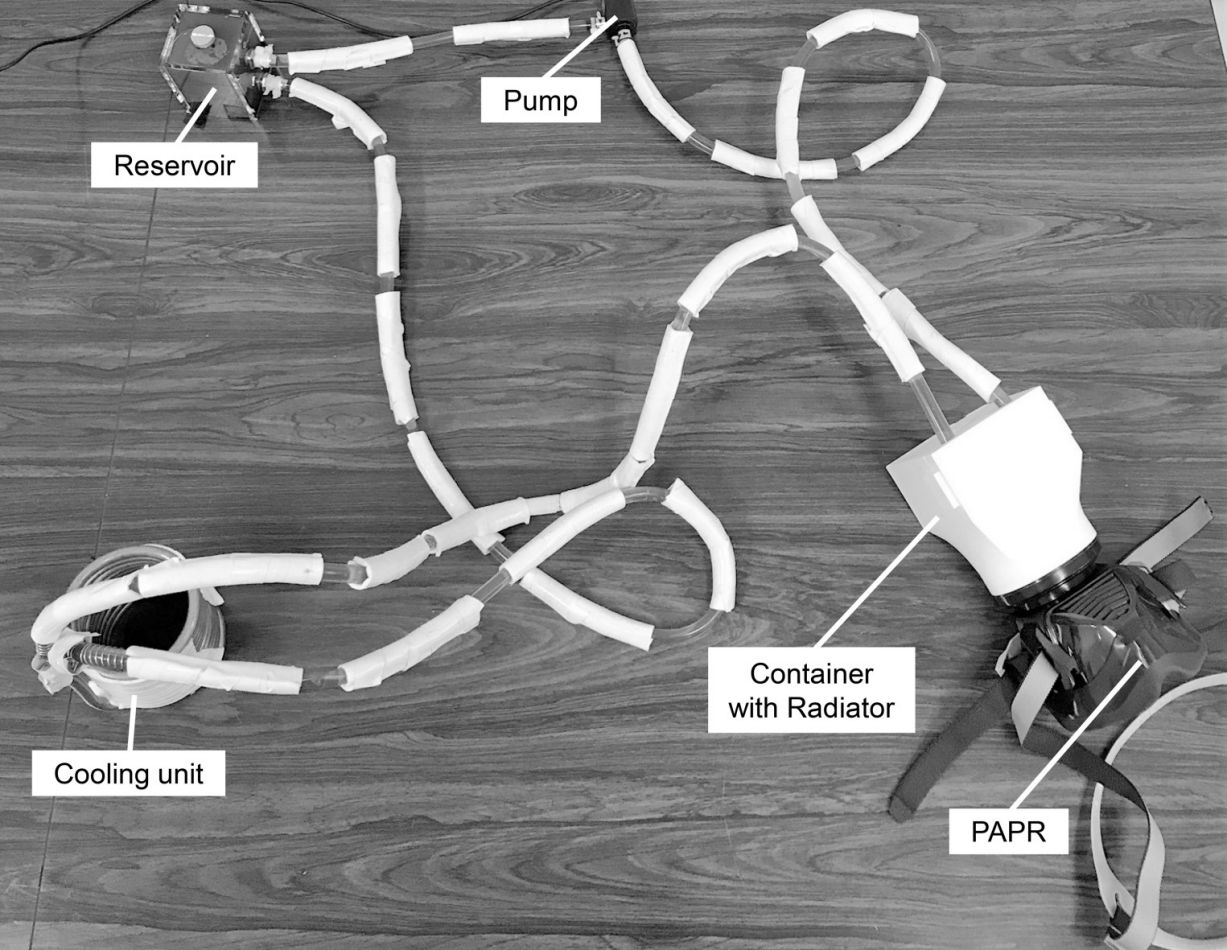
Photograph of the cooling device. PAPR: powered air-purifying respirator.

This cooling device consisted of a liquid-cooling system. The antifreeze (ethylene glycol) cooled in the cooling unit was circulated by the pump and passed through the radiator to cool the surrounding air. The pump used for this device was DC30A-1230 (Shenzhen Zhongke Century Technology Co., Ltd., China), and the radiator used for this device was BU0355 (GAOHOU, China). The cooling unit consisted of one hollow, spiral-shaped copper tube. The antifreeze that passed through the copper tube was cooled by attaching the refrigerant inside and outside the copper tube. A reservoir was inserted into the circuit to replenish the antifreeze. The cooling unit, radiator, pump, and reservoir were connected using hollow plastic tubes with heat insulation tape. Furthermore, we made the original container so that the radiator could be sealed close to the PAPR inlet. The container was modeled using 3D CAD (Fusion360, Autodesk Inc., USA) and printed using a 3D printer (Creator3, Zhejiang Flashforge3D technology Co., LTD., China). Acrylonitrile butadiene styrene (ABS) was used as the container material. A diagram of the cooling device is shown in Fig 1, and a photograph is shown in Fig 2.

As shown in Fig 2, the cooling unit, radiator, pump, and reservoir were connected using hollow plastic tubes. The antifreeze circulated the cooling unit, radiator, pump, and reservoir in this order. The ambient air was cooled as it passed through the radiator.

### 2.5 Measurement of the workloads at the maximal oxygen uptake

Before the day of the experiment (measurement of temperature), the workloads at the maximal oxygen uptake (VO_2max_) were measured for each subject.

The measurements were conducted using open-circuit spirometry (ARCO-2000; ARCO SYSTEM INC., Japan) and a cycle ergometer (Health Guard ACTIVE 10 III, Takei Scientific Instruments Co., Ltd, Japan) in an artificial climate chamber (TBR-8E20W0P2T, ESPEC CORP., Japan) set at a dry-bulb temperature of 25°C and relative humidity of 50%. The measurements were performed by arranging the procedure defined by the American College of Sports Medicine [27].

The measurements were conducted at different levels of workload powers (W or m^2^·kg/s^3^). The subjects sat on a cycle ergometer and rested for 2 min. They then began exercising at 20 W and the workload was increased by 10 W every minute. During measurement, their heart rates (HRs) were monitored using a heart rate monitor (BSM-2401, NIHON KOHDEN CORPORATION, Japan). Tests were terminated at workload up to the point where 80% of the target heart rate (80% THR) was achieved or at workload up to the point where the perceived exertion rating monitored by the Borg category was at its maximum. The 80% THR of each subject was calculated using the following formula:

$$80\%THR=0.8\times ({HR}_{max}-{HR}_{rest})+{HR}_{rest}$$

where HR_rest_ is the resting HR of each subject. HR_max_ was calculated using the following formula.


$${HR}_{max}=208-0.7\times age\;of\;each\;subject\;(year)$$

**Fig 3:**
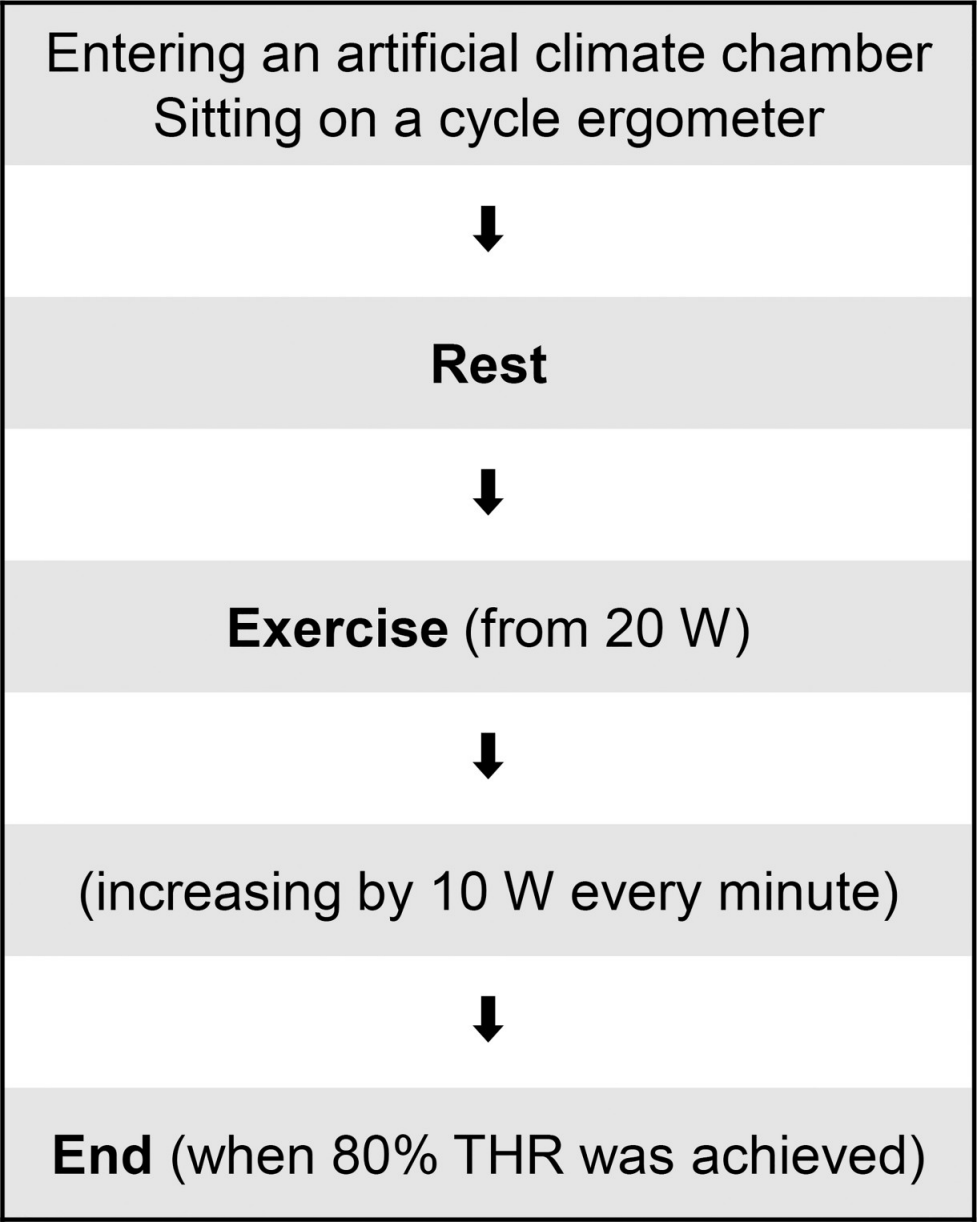
Outline of the experiment for the measurement of the workloads at the maximal oxygen uptake. 80% THR: 80% of the target heart rate.

The outline of the experiment is shown in Fig 3.

During exercise, oxygen uptake, and HR were measured every 10 s. Notably, the mean oxygen uptake and HR after 40 and 50 s had elapsed during exercise at each workload were defined as the oxygen uptake and HR at that workload. We calculated the oxygen uptake and HR for each workload from 40 W to the workload at the end of the exercise.

The value of oxygen uptake corresponding to the value of HR_max_ calculated using the abovementioned equation (VO_2max_) was measured from the regression line obtained from “oxygen uptake from 40 W to workload at the end of the measurement” and “HR from 40 W to workload at the end of the measurement.” Then, the value of workloads corresponding to the value of VO_2max_ calculated by the abovementioned method (workload at VO_2max_) was calculated from the regression line obtained from “workloads from 40 W to the end of the measurement” and “oxygen uptake from 40 W to workload at the end of the measurement.” The regression line was calculated using Microsoft Excel 2016 MSO (16.0.14228.20216) (Microsoft Corporation, USA).

The mean VO_2max_ was 38.9±7.7 ml/kg/min and the mean workload at VO_2max_ was 187.4±29.1 W.

### 2.6 Experimental procedures

A crossover study was conducted applying three sets of conditions in a randomly assigned order involving 10 subjects. The subjects wore either a PAPR equipped with a cooling device (C-PAPR), PAPR, or RPR for the experiment. The experiment was conducted three times per subject, each on a different day.

**Fig 4:**
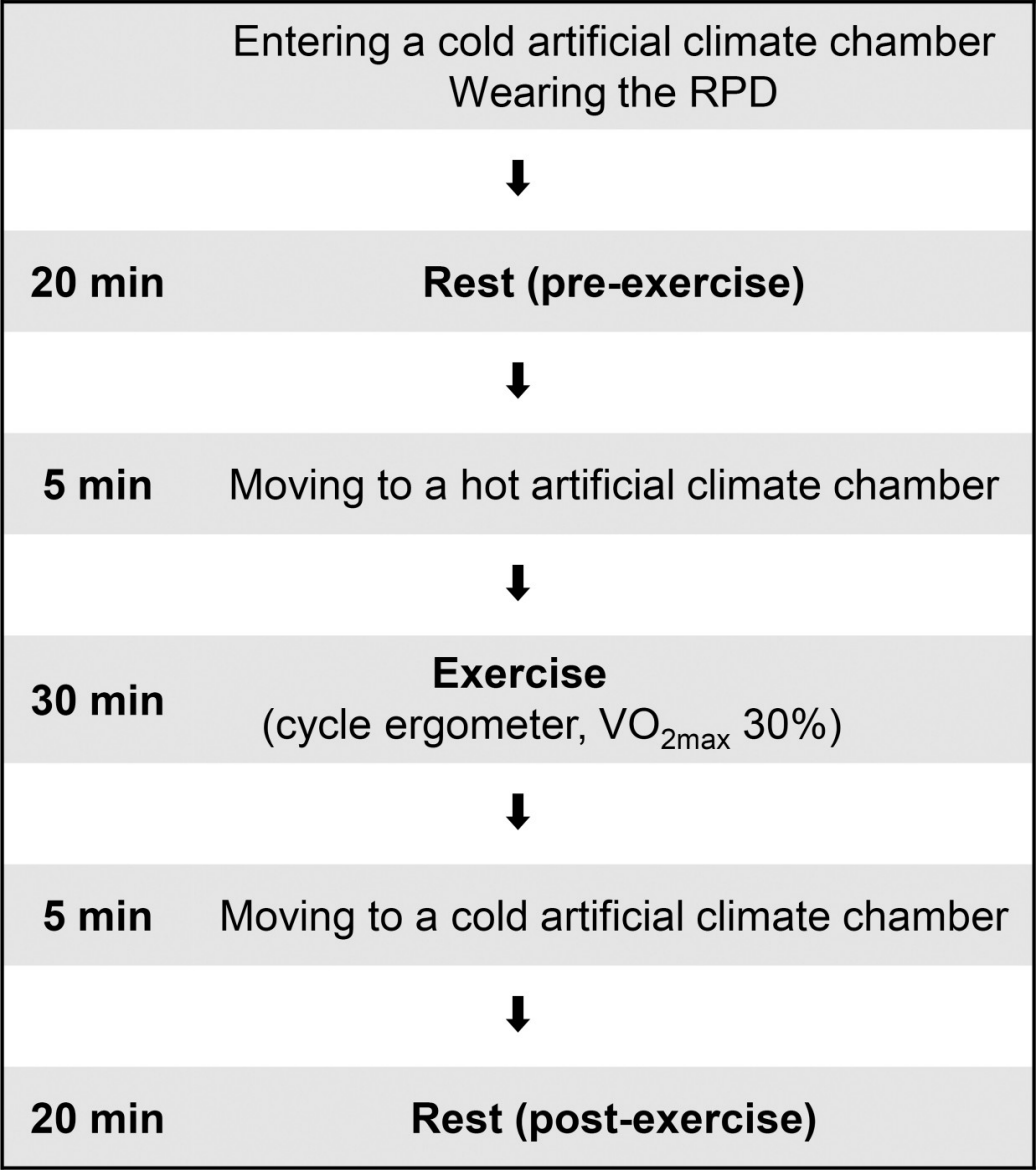
Outline of the experiment for the measurement of temperature. Cold artificial climate chamber: dry-bulb temperature of 28°C and relative humidity of 45%, RPD: respiratory protective device; hot artificial climate chamber: dry-bulb temperature 36°C and relative humidity 45%; VO_2max_: maximal oxygen uptake.

**Fig 5:**
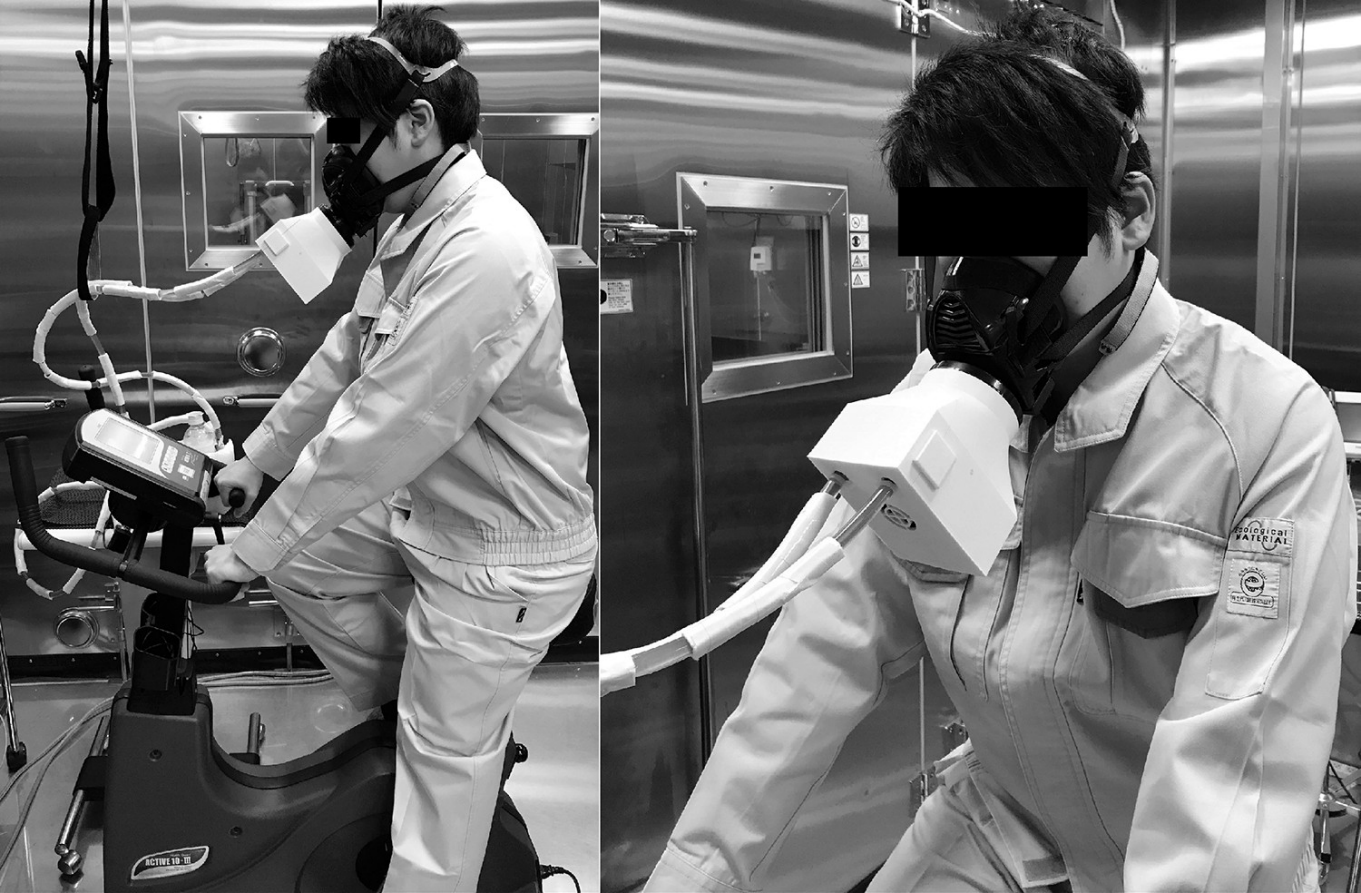
Photograph of the experiment for the measurement of temperature. The subject exercised using the cycle ergometer in a hot artificial climate chamber set at a dry-bulb temperature of 36°C and relative humidity of 45%. The image shows a subject wearing a powered air-purifying respirator equipped with a cooling device.

The procedure of the experiment was as follows. The subject changed into long-sleeved and long-pant work clothes. To minimize the influence of clothing, all subjects wore the same type of clothing. Then, the subject entered a cold artificial climate chamber set at a dry-bulb temperature of 28°C and relative humidity of 45%. The subject self-inserted a rectal temperature probe (copper-constantan thermocouple, 4 mm in diameter) approximately 150 mm from the anal sphincter. The subjects then wore the RPD (either C-PAPR, PAPR, or RPR). When the C-PAPR was worn, a thermometer was inserted near the PAPR inlet in the container of the cooling device to avoid contact with the PAPR, container, or skin. After these preparations, the subject rested in a cold artificial climate chamber for 20 min. The subject then moved to a hot artificial climate chamber set at a dry-bulb temperature of 36°C (relative humidity of 45%) in 5 min and sat on a cycle ergometer. The subject exercised at 30% of the workload at VO_2max_ using a cycle ergometer for 30 min. After the exercise, the subjects moved to the cold artificial climate chamber in 5 min, where they rested for 20 min. Measurements were obtained continuously for a total of 80 min from pre-exercise rest to post-exercise rest. The outline of the experiment is shown in Fig 4, and a photograph of the experiment is shown in Fig 5.

### 2.7 Measurement of temperature

During the experiment, the rectal temperature of the subjects was continuously measured as a representative core temperature. The temperature near the PAPR inlet in the container of the cooling device was continuously measured when the C-PAPR was worn. These temperatures were measured and recorded every 10 s (0, 10, 20, 30, 40, and 50 s of each minute) using a data logger (NR-500, KEYENCE CORPORATION, Japan). The mean rectal temperature every 10 s (0, 10, 20, 30, 40, and 50 s of each minute) during each minute was defined as the rectal temperature during that minute. For example, the rectal temperature at “minute 1” is the mean rectal temperature at 1 min 0 s, 1 min 10 s, 1 min 20 s, 1 min 30 s, 1 min 40 s, and 1 min 50 s. Similarly, the mean temperature near the PAPR inlet in the container of the cooling device every 10 s (0, 10, 20, 30, 40, and 50 s of each minute) during each minute was defined as the temperature near the PAPR inlet in the container of the cooling device during that minute. The rectal temperature was calculated as the difference between the value at minute 0 and the temperature at the studied minute.

### 2.8 Statistical methods

The repeatedly measured rectal temperature data were nested into seven experimental phases, excluding the time to move to the next room: early-pre-exercise time (0–9 min), late-pre-exercise time (10–19 min), early-during-exercise time (25–34 min), mid-during-exercise time (35–44 min), late-during-exercise time (45–54 min), early-post-exercise time (60–69 min), and late-post-exercise time (70–79 min). The repeated data were analyzed using a linear mixed model (LMM), with rectal temperature as the outcome variable. Among the predictor variables, the fixed factors were the types of RPD, seven experimental phases, and interaction of the types of RPD with respect to the seven experimental phases; the random factor was the survey participants. When any significant main effect or interaction effect was detected, a multiple comparison test adjusted using the Bonferroni method was performed for individual experimental phases as a post hoc test. The statistical significance was set at *p* <0.05. All statistical analyses were conducted using IBM SPSS Statistics (version 23.0, IBM Co., USA).

### 2.9 Ethics approval

This study was approved by the Ethics Committee of Medical Research of the University of Occupational and Environmental Health, Japan (receipt number: R2-016, August 22th, 2019). Written informed consent was obtained from all participants before starting the experiment.

## 3. Results

### 3.1 Inhalation temperature

**Fig 6:**
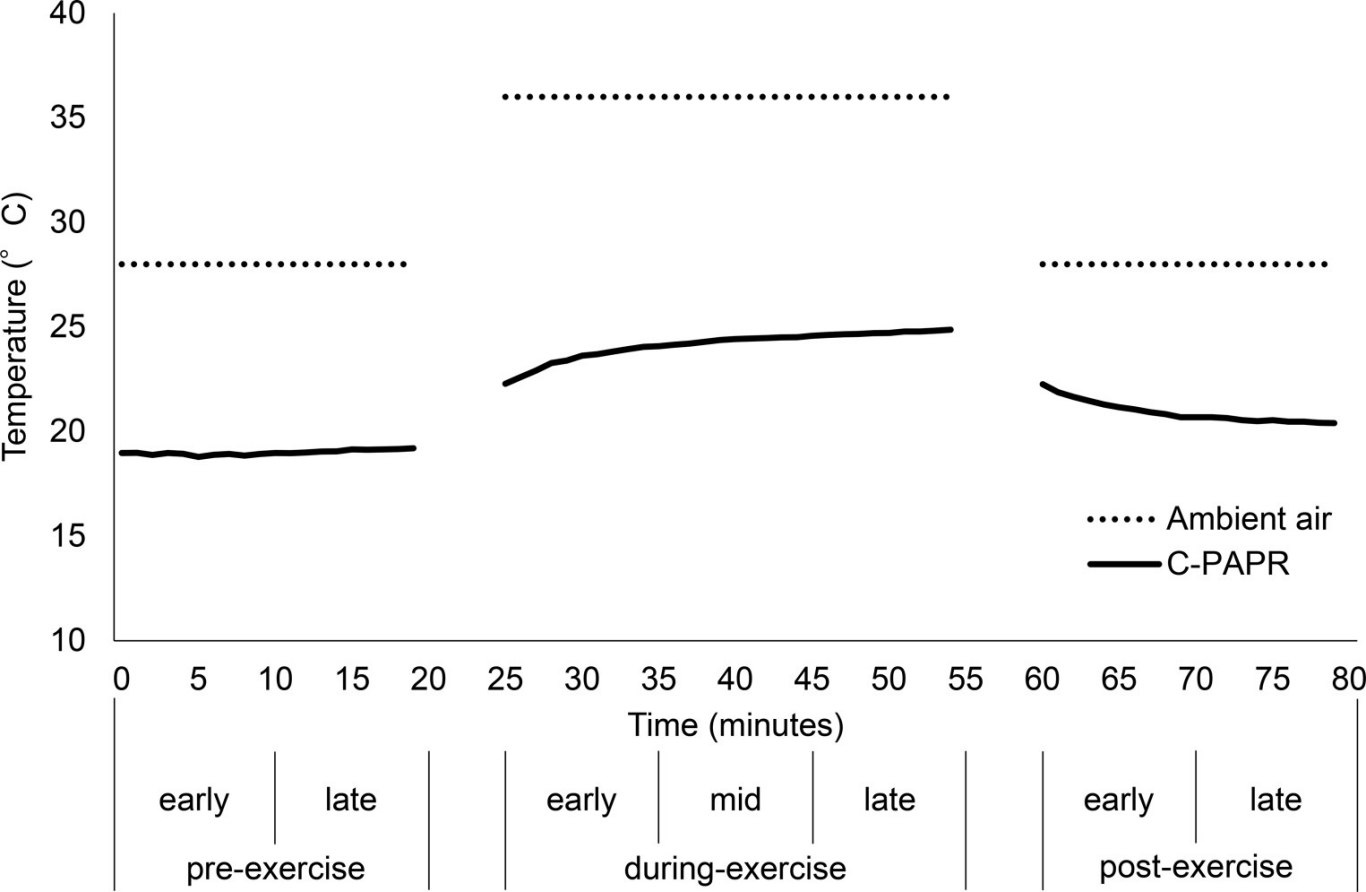
Inhalation temperature. The ambient air temperature and temperature near the powered air-purifying respirator inlet in the container of the cooling device were measured. The measured values of the time taken to move to the next room were excluded; C-PAPR: temperature near the powered air-purifying respirator inlet in the container of the cooling device.

The measurements of the ambient air temperature and temperature near the PAPR inlet in the container of the cooling device when the C-PAPR was worn (mean of 10 subjects) are shown in Table 1 and are plotted in Fig 6. The difference between the ambient air temperature and temperature near the PAPR inlet in the container of the cooling device when the C-PAPR was worn was as follows: pre-exercise, at least 8.80°C; during-exercise, 11.13°C; and post-exercise, 5.74°C.

**Table 1:** Inhalation temperature.

		Pre-exercise		During-exercise			Post-exercise	
		Early	Late	Early	Mid	Late	Early	Late
**Ambient air** (°C)		28		36			28	
**C-PAPR**	mean (°C)	18.91	19.08	23.36	24.34	24.72	21.32	20.54
	95% CI	18.87–18.96	19.02–19.14	22.94–23.78	24.23–24.46	24.65–24.79	20.97–21.68	20.47–20.61

C-PAPR, temperature near the powered air-purifying respirator inlet in the container of the cooling device; 95% CI, 95% confidence interval.

### 3.2 Rectal temperature

**Fig 7:**
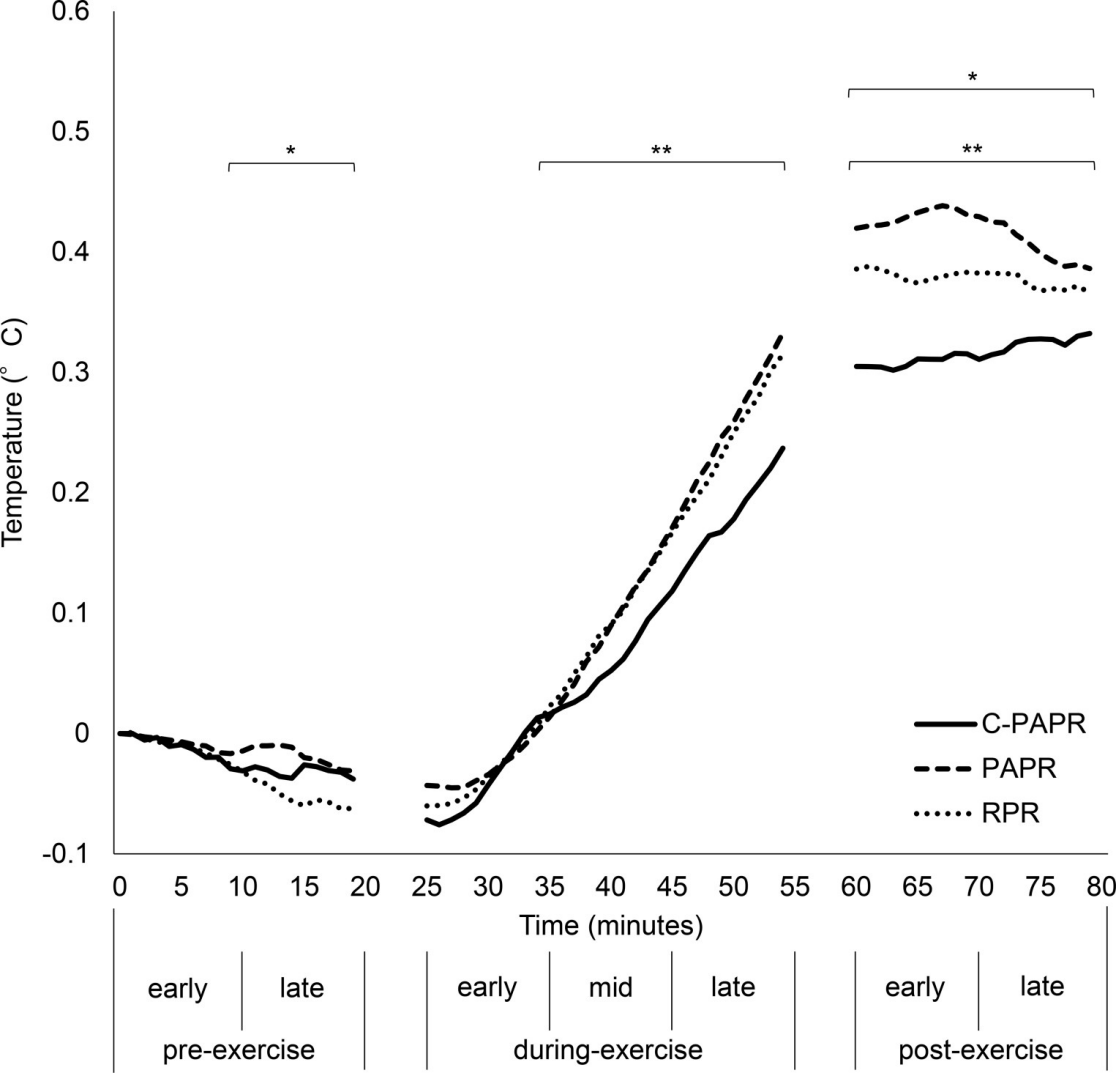
Rectal temperature. The temperature was calculated as the difference between the temperature value at minute 0 and at the minute studied. The measurement values for the time taken to move to the next room were excluded. *Temperature of the RPR was significantly lower than that of PAPR, **temperature of C-PAPR was significantly lower than that of PAPR and RPR. C-PAPR: powered air-purifying respirator equipped with cooling device; PAPR: powered air-purifying respirator; RPR: replaceable particulate respirator.

The rectal temperatures (calculated as the difference between the temperature value at minute 0 and at the minute studied) of each RPD (mean of 10 subjects) are shown in Table 2 and are plotted in Fig 7. The difference between the rectal temperature of C-PAPR and that of PAPR was a maximum of 0.10°C during exercise and 0.13°C post-exercise. Similarly, the difference between the rectal temperature of C-PAPR and that of RPR was a maximum of 0.08°C during exercise and 0.08°C post-exercise.

**Table 2:** Rectal temperature.

		Pre-Exercise			During Exercise					Post-Exercise			
		Early	Late		Early	Mid		Late		Early		Late	
**C-PAPR**	mean (°C)	-0.01	-0.03		-0.04	0.05	^‡^*p* = 0.037 ^§^*p* = 0.017	0.18	^‡^*p*<0.001 ^§^*p*<0.001	0.31	^‡^*p*<0.001 ^§^*p*<0.001	0.32	^‡^*p*<0.001 ^§^*p*<0.001
	95% CI	-0.02–0.00	-0.03–-0.03		-0.06–-0.02	0.03–0.08		0.15–0.20		0.31–0.31		0.32–0.33	
**PAPR**	mean (°C)	-0.01	-0.02		-0.03	0.08		0.25		0.43		0.41	
	95% CI	-0.01–0.00	-0.02–-0.01		-0.04–-0.02	0.05–0.12		0.21–0.29		0.42–0.43		0.39–0.42	
**RPR**	mean (°C)	-0.01	-0.05	^†^*p* = 0.012	-0.04	0.09		0.24		0.38	^†^*p*<0.001	0.37	^†^*p* = 0.020
	95% CI	-0.02–0.00	-0.06–-0.04		-0.05–-0.02	0.05–0.12		0.20–0.28		0.38–0.38		0.37–0.38	

^†^Temperature of the RPR was significantly lower than that of PAPR

^‡^temperature of C-PAPR was significantly lower than that of PAPR

^§^temperature of C-PAPR was significantly lower than that of RPR. C-PAPR, powered air-purifying respirator equipped with a cooling device; PAPR, powered air-purifying respirator; RPR, replaceable particulate respirator; 95% CI, 95% confidence interval.

The types of RPDs significantly affected rectal temperature (*p* <0.001). Additionally, we compared the rectal temperatures among the different RPDs. During the late pre-exercise period, the rectal temperature of the subjects with the RPR was significantly lower than that of the subjects with the PAPR. After the mid-during-exercise period, the rectal temperature of the subjects with the C-PAPR was significantly lower than that of the subjects with the PAPR. Furthermore, after the mid-during-exercise period, the rectal temperature of the subjects with the C-PAPR was significantly lower than that of the subjects with the RPR. After the early-post-exercise period, the rectal temperature of the subjects with the RPR was significantly lower than that of the subjects with the PAPR. Other combinations were not statistically significant.

## 4. Discussion

The temperature near the PAPR inlet in the container of the cooling device was approximately 10°C lower than the ambient air temperature. The subject inhaled air whose temperature was also considered to be approximately 10°C lower than that of the ambient air. Previous studies on internal cooling by cold-air inhalation showed that the temperature of the air inhaled by the subjects was 3.6°C and 10°C, being 34.4°C and 28°C lower than the ambient air temperature, respectively [16, 17]. In the present work, it was necessary to achieve both cooling performance and device miniaturization for future practical applications (possibly in a real working environment). Because of this, and owing to the technical limitations of the cooling device, the temperature of the inhalation air was higher than that in previous studies. However, the PAPR equipped with the cooling device abated the rise in the deep-body temperature after the mid-during-exercise time.

Body heat balance can be explained as follows [28, 29]:

$$S=M-W-(C+R+E_{sk})+(C_{res}+E_{res})$$

where S is the rate of heat storage (W/m^2^), M is the rate of metabolic energy production, W is the rate of mechanical work of the body, C, R, and E_sk_ refer to the rate of convective heat exchange, rate of radiative heat exchange, and rate of evaporative heat exchange from the skin, respectively. Finally, C_res_ is the rate of convective heat exchange from respiration, and E_res_ is the rate of evaporative heat exchange from respiration. There are two main components of heat exchange through respiration: first, convective heat exchange as a function of cool air inhalation, where, heat from the lungs is transferred in exhalation (C_res_), and second, evaporative heat exchange as a function of moisture saturation in the exhaled air (E_res_). Although a mechanism to find the proportion of heat exchange through each component of the respiratory heat exchange has yet to be determined, under normal conditions, the total amount of respiratory heat exchange as a function of C_res_ and E_res_ is 10–15 W. This accounts for 10% of the body’s total heat exchange [30]. In this study, it is thought that cold air inhalation by the cooling device abated the rise in deep body temperature. In addition, previous studies revealed that subjects did not report any incidences of respiratory distress from breathing cold air (3.6°C), and it did not pose any danger or cause damage to the respiratory tract [16].

In Japan, the type and performance standard of PAPRs is defined by the Ministry of Health, Labour and Welfare [25]. Because our cooling device does not modify the respirator or filter itself, even if it is attached to the PAPR, it would not affect the performance of the respirator or violate the aforementioned standard. Furthermore, with adjustment of the container, it can be attached to other types of PAPRs (tight fitted with a half facepiece). However, this cooling device is a prototype, and there is room for improvement. For example, further miniaturization (improved mobility and reduction of physical load on the user) and increasing the robustness of the entire cooling device can be explored in the future.

High protection performance of PAPRs has already been reported in Japan [31–33], and The Ministry of Health, Labour and Welfare’s 2018 report, the Ninth Comprehensive Measures to Prevent Hazards Due to Dust, recommends the use of PAPR [34]. However, excluding applications that require the exclusive use of PAPR, only a few workers use such respirators [19]. Over the years, various disadvantages of PAPR have been reported, such as decreased communication ability, mobility, and dexterity [20]. As mentioned above, workers engaged in dust-generating scenarios are often subjected to the risk of heat stress. In these situations, it is important to take measures to prevent not only pneumoconiosis but also heatstroke. Therefore, we conclude that the advantages of using a PAPR in hot environments outweigh its disadvantages and may save lives.

In this study, there were periods during which the rectal temperatures of participants using the PAPR (without a cooling device) were higher than of those using the RPR. While the direct contribution of the RPD to metabolic cost is considered to be minor [35], the impact it has on body temperature is likely to increase with longer, uninterrupted periods of use at high ambient temperatures and humidity and higher work rates [35]. Results show that using a PAPR (without a cooling device), particularly in very hot environments, may increase the body temperature.

There were several limitations to this study. First, we only used one type of PAPR and RPR. Though the effect of this scenario has not been verified, it is possible to attach the cooling device to other types of PAPRs by adjusting or creating a container. Hence, extending our analysis to multiple types of RPDs is a potential future direction for related studies. Second, over the course of the study, only one pattern of temperature and humidity settings and exercise and rest periods were applied. However, practically, the temperature and humidity in actual workplaces vary, as do the working and resting hours. Therefore, the future scope of our study could consider multiple types of conditions that mimic real-life situations.

## 5. Conclusions

In this study, we have successfully verified that internal cooling using a tight-fitting half-facepiece breath-response PAPR equipped with a self-produced cooling device abated an increase in the deep body temperature while performing activities. PAPR is useful because of its potential application in hot occupational environments and can save lives in working environments where heat stress can result in major medical complications.
